# Investigation on the Mental Health Status of ICU Practitioners and Analysis of Influencing Factors During the Stable Stage of COVID-19 Epidemic in China

**DOI:** 10.3389/fpubh.2021.572415

**Published:** 2021-08-18

**Authors:** Wei He, Wenjin Chen, Xiaopeng Li, Sui Sum Kung, Liangnan Zeng, Tangming Peng, Xiaomeng Wang, Reng Ren, Di Zhao

**Affiliations:** ^1^Department of Critical Care Medicine, Beijing Tongren Hospital, Capital Medical University, Beijing, China; ^2^Department of Neurosurgery, Xuanwu Hospital, Capital Medical University, Beijing, China; ^3^Department of Intensive Care Unit, The First Affiliated Hospital of Xinjiang Medical University, Urumqi, China; ^4^Division of Neurosurgery, Department of Surgery, Kaohsiung Medical University Hospital, Kaohsiung Medical University, Kaohsiung, China; ^5^Department of Neurosurgery, Affiliated Hospital of Southwest Medical University, Chengdu, China; ^6^Emergency Intensive Care Unit, Xuzhou Central Hospital, Xuzhou, China; ^7^Neurocritical Care Unit, Second Affiliated Hospital of Zhejiang University School of Medicine, Hangzhou, China; ^8^Department of Neurosurgery, The First Hospital of Hebei Medical University, Shijiazhuang, China

**Keywords:** COVID-19, ICU practitioners, mental health, SCL-90, intervening measure

## Abstract

**Objective:** To understand the impact of COVID-19 epidemic on the mental health status of intensive care unit (ICU) practitioners in China, and to explore the relevant factors that may affect the mental health status of front-line medical workers so as to adopt efficient and comprehensive measures in a timely manner to protect the mental health of medical staff.

**Methods:** The study covered most of the provinces in China, and a questionnaire survey was conducted based on the WeChat platform and the Wenjuanxing online survey tool. With the method of anonymous investigation, we chose ICU practitioners to participate in the investigation from April 5, 2020 to April 7, 2020. The respondents were divided into two groups according to strict criteria of inclusion and exclusion, those who participated in the rescue work of COVID-19 (COVID-19 group) and those who did not (non-COVID-19 group). The SCL-90 self-evaluation scale was used for the evaluation of mental health status of the subjects.

**Results:** A total of 3,851 respondents completed the questionnaire. First, the overall mental health status of the targeted population, compared with the Chinese norm (*n* = 1,388), was reflected in nine related factor groups of the SCL-90 scale, and significant differences were found in every factor in both men and women, except for the interpersonal sensitivity in men. Second, the overall mental health of the non-COVID-19 group was worse than that of the COVID-19 group by the SCL-90 scale (OR = 1.98, 95% CI, 1.682–2.331). Third, we have revealed several influencing factors for their mental health in the COVID-19 group, current working status (*P* < 0.001), satisfaction of diet and accommodation (*P* < 0.05), occupational exposure (*P* = 0.005), views on the risk of infection (*P* = 0.034), and support of training (*P* = 0.01).

**Conclusion:** The mental health status of the ICU practitioners in the COVID-19 group is better than that of the non-COVID-19 group, which could be attributed to a strengthened mentality and awareness of risks related to occupational exposure and enforced education on preventive measures for infectious diseases, before being on duty.

## Introduction

The year 2020 was disrupted by a sudden pandemic outbreak of the coronavirus disease 2019 (COVID-19), which was first reported in Wuhan, China (1), and it is becoming an emerging, rapidly evolving situation. According to the official website of WHO, over 160 million people have been confirmed to have a COVID-19 infection globally by the end of May 25, 2021 (2). Until now, COVID-19 is still raging in much of the world as global cases hit record highs. We have accumulated much knowledge about the COVID-19, including the virus information, clinical features, and diagnosis, but there is no effective treatment (3–5). There is extreme fear over the COVID-19 among the general public because of the strong infectivity, fast transmission, and non-specific manifestations (6). Harsh protective measures have been put in force in real-life practice. Surprisingly, surveys after the outbreak of epidemic have found that most patients who were diagnosed usually have only mild pain or moderate mental problems, including depression (DEP), anxiety (ANX), stigma, and sorrow (7). However, medical health workers are the front-line fighters to treat COVID-19 patients, facing a high risk of infection every day. In order to combat the outbreak, they need to work overtime under a stressful mentality. In short, they are under a kind of enduring pressure that risks to exceed their coping ability (8). Everyone knows that attention should be paid to the mental health of medical workers during the fight against COVID-19 (9, 10), and some reports have been made on the mental health of medical workers after the outbreak of COVID-19 in China. Zhang et al. conducted a survey on the psychosocial problems of both medical and non-medical health workers during the COVID-19 outbreak (11). They found that medical health workers had psychosocial problems and risk factors for developing them. A report conducted in Wuhan found that poor mental status and sleep quality were common among frontline healthcare workers during the COVID-19 outbreak (12). However, there are some limitations in the sample size and sampling representativeness of these studies. Hence, in this study, we aim to understand the impact of COVID-19 epidemic on the mental health status of intensive care unit (ICU) practitioners nationally and to explore the relevant factors that may affect the mental health status of front-line medical workers so as to take measures to protect the mental health of medical staff as quickly and comprehensively as possible.

## Methods

### Study Design

This study was a cross-sectional online survey performed based on WeChat platform and Wenjuanxing (a platform providing functions equivalent to Amazon Mechanical Turk) from April 5 to April 7, 2020, which basically was the stable stage of COVID-19 epidemic in China. This study was approved by the Medical Ethics Committee of The First Hospital of Hebei Medical University (No. 20200211). The informed consent part was at the front of the questionnaire, and the participants who received the questionnaire had the right to refuse to answer.

### Study Population

In China, the usage rate of WeChat could reach 100% medical staff in almost every department as each unit has its own WeChat group. A lot of information about departments and hospitals was sent through the WeChat group, and the questionnaires were distributed to the working group by the directors of the provincial and local hospitals. All the subjects were essential medicine practitioners, and there were no non-professionals among them. However, some people may be unwilling to fill in the questionnaire; besides, the representative sample covered the whole country and could provide relevant and supportive data. Third, the questionnaire was based on a Wenjuanxing platform and distributed by WeChat. Quality control falls into consideration since program design that participants are required to answer all the questions. Meanwhile, Wenjuanxing could set a time-frame in case of answering the questionnaire. This study had a preliminary investigation phase (small sample), when the authors evaluated the reasonable time setting of answering the questionnaire, but was not recorded in the final statistics (i.e., answers made out of the opening hours would be ruled out), the survey of COVID-19 would expire over time, and the sample size collected during the opening hour had reached the expectation.

With the method of anonymous investigation, ICU practitioners from most of the provinces in China were recruited in the study. The respondents who completed all questions of the online survey were divided into two groups according to strict criteria of inclusion and exclusion—those who participated in the rescue work of COVID-19 (anti-COVID-19 group) and those who did not (non-anti-COVID-19 group).

Inclusion criteria included the following: (a) Critical care medical practitioners, including doctors, nurses, and respiratory therapists; (b) Personnel in China, including 23 provinces, five autonomous regions, and four municipalities directly under the central government, excluding two special administrative regions of Hong Kong and Macau; (c) In-service personnel.

Exclusion criteria included (d) Those who did not answer the questionnaire in the opening hours; (e) Exceeding the time limit for questionnaire; (f) incomplete answering of the questionnaire.

### Measurements

Information collected *via* survey questions were demographic data, i.e., gender, age, occupation (doctors, nurses, and others), education status (community college, bachelor, master, and doctor), marital status (married, unmarried, and other), professional title, department (ICU, surgical department, internal medicine, pneumology department, etc.), medical working time, having siblings or children, religious belief, having participated in public health emergency treatment before or not, and directly participating in COVID-19 antiepidemic work or not. Symptom check list-90 (SCL-90) (13) was used to check the mental health status of the subjects, including somatization (SOM), obsessive–compulsive (OC), interpersonal sensitivity (IS), DEP, ANX, hostility (HOS), phobic anxiety (PHOB), paranoid ideation, and psychoticism (PSY). It is a 90-item self-report scale with items to be rated on a five-point Likert scale (from 1 “not at all” to 5 “extremely”).

### Outcome Definition

The statistical index of SCL-90 mainly consists of two indicators, namely total score (score range: 90–450) and factor score (score range: 1–5). Any subscale score of all above nine factors ≥2 indicate potential psychological issues (14). The number of positive items referred to the number left in the 90 questions at excluding “No” answers (score ≥2). Number of negative items referred to the number of items with a score of 1, indicating the subject does not experience the symptom. A positive result is the total score of SCL-90 ≥160 or positive items >43.

### Reliability and Validity

Internal consistency of the SCL-90 score (Chinese version) was assessed by Cronbach's α and an average interitem correlation. Cronbach's α for summary score of 0.70–0.80 is considered satisfactory for a reliable comparison between groups, and more than 0.90 is required for clinical usefulness of the instrument (15).

### Statistical Analysis

The respondents who completed all questions of the online survey were divided into two groups according to strict criteria of inclusion and exclusion. They are those who participated in the rescue work of COVID-19 (anti-COVID-19 group) and those who did not (non-anti-COVID-19 group). The measurement variables were expressed as mean ± standard deviation (SD), and by *U*-test the scores of SCL-90 factors of the ICU practitioners were compared with the Chinese norm, which is currently used in China and translated by Dang et al. (16). Frequency (%) was used for counting variables, and the Chi-square or Fisher method was used for intergroup comparison. Logistic multivariate regression was used to analyze the influence factors of the positive symptom of SCL-90 score, and the OR value was estimated. In the multivariate analysis, all features of the subjects were forced to be included in the model as independent variables, and on this basis, stepwise regression was carried out. The probability value of the stepwise regression in the setting of independent variables inclusion and removal was 0.05. The software used for statistical analysis was SAS 9.3. Both groups were tested bilaterally. When *P* < 0.05, the difference was considered statistically significant.

### Patient and Public Involvement

There was no patient or public involvement in the design and conduct of the study.

## Results

### General Characteristics of ICU Practitioners During COVID-19 Epidemic

A total of 3,851 ICU practitioners [1,527 nurses (39.65%) and 2,324 doctors (60.35%)] participated in this questionnaire survey. Most of them were from the ICU (74.68%). Out of the total, 1,210 (31.42) people were directly involved in the fight against the COVID-19 epidemic. The age, educational background, professional title, marriage, and other general characteristics of the respondents are shown in Table 1.

**Table 1:** SCL-90 score of ICU practitioners.

**Factor**	**Overall**			**Male**			**Female**		
	**ICU practitioners**	**Norm**	p^*^	**ICU practitioners**	**Norm**	p^*^	**ICU practitioners**	**Norm**	p^*^
Somatization	1.60 ± 0.66	1.37 ± 0.48	<0.001	1.53 ± 0.6	1.38 ± 0.49	<0.001	1.65 ± 0.70	1.37 ± 0.47	<0.001
Obsessive-compulsive s	1.91 ± 0.79	1.62 ± 0.58	<0.001	1.84 ± 0.76	1.66 ± 0.61	<0.001	1.96 ± 0.81	1.59 ± 0.54	<0.001
Interpersonal sensitivity	1.69 ± 0.74	1.65 ± 0.61	0.048	1.67 ± 0.72	1.66 ± 0.64	0.735	1.71 ± 0.75	1.61 ± 0.58	<0.001
Depression	1.75 ± 0.75	1.50 ± 0.59	<0.001	1.70 ± 0.72	1.51 ± 0.6	<0.001	1.78 ± 0.78	1.49 ± 0.56	<0.001
Anxiety	1.61 ± 0.70	1.39 ± 0.43	<0.001	1.58 ± 0.67	1.41 ± 0.44	<0.001	1.63 ± 0.71	1.37 ± 0.42	<0.001
Hostility	1.67 ± 0.76	1.46 ± 0.55	<0.001	1.65 ± 0.74	1.48 ± 0.56	<0.001	1.68 ± 0.77	1.45 ± 0.52	<0.001
Phobic anxiety	1.41 ± 0.60	1.23 ± 0.41	<0.001	1.37 ± 0.55	1.23 ± 0.37	<0.001	1.44 ± 0.63	1.30 ± 0.47	<0.001
Paranoid ideation	1.54 ± 0.67	1.43 ± 0.57	<0.001	1.55 ± 0.67	1.46 ± 0.59	0.001	1.52 ± 0.66	1.41 ± 0.54	<0.001
Psychoticism	1.47 ± 0.63	1.29 ± 0.42	<0.001	1.46 ± 0.61	1.32 ± 0.44	<0.001	1.48 ± 0.64	1.26 ± 0.39	<0.001
Number of positive items	34.57 ± 27.9	24.92 ± 18.41	<0.001	34.12 ± 28.31	25.68 ± 18.79	<0.001	34.91 ± 27.59	24.17 ± 17.49	<0.001

* *Comparison between ICU practitioners and Chinese norm*.

Table 2 showed the current working status of the workers directly participating in the fight against the COVID-19 epidemic. There were 995 (82.23%) who had finished the anti-COVID-19 work and had been in the succeeding period or back to work, and the other 215 (17.77%) were still in the rescue work. About two-thirds of the participants had worked for more than a month in the fight against the COVID-19 epidemic. More than half of the people were satisfied with their diet and accommodation during the epidemic, whereas only a minority (2.23–3.22%) was dissatisfied. About 65.45% believed that the training they had received in the prevention and treatment of infectious diseases was adequate in both theory and practice. In comparison, a minority (1.40%) believed that the theory was inadequate and poor in operability. Moreover, the proportion who thought they were at high risk of infection at work reached 43.88%. The proportions of suspicious occupational exposure and infection caused by occupational exposure were 36.61 and 9.42%, respectively. During the antiepidemic period, the weekly working hours were generally substantial, with about half of the staff working more than 40 h per week, and 8.02% working more than 80 h.

**Table 2:** The positive symptom ratio of SCL-90 score in ICU practitioners with different characteristics.

**Variable**	**Variable level**	**Positive symptom, *n* = 1,251 (%)**	**Negative symptom, *n* = 2,600 (%)**	**Test method**	**Statistics (χ^**2**^)**	***p***
Gender	Male	512 (30.59)	1,162 (69.41)	Chi square	4.872	0.027
	Female	739 (33.95)	1,438 (66.05)			
Age	≤ 25	75 (33.48)	149 (66.52)	Chi square	5.716	0.221
	26–30	220 (32.45)	458 (67.55)			
	31–35	333 (34.62)	629 (65.38)			
	36–40	265 (33.38)	529 (66.62)			
	>40	358 (30.01)	835 (69.99)			
Highest education	Community college	77 (26.83)	210 (73.17)	Chi square	22.230	<0.001
	Bachelor	894 (34.80)	1,675 (65.20)			
	Master	247 (29.27)	597 (70.73)			
	Doctor	33 (21.85)	118 (78.15)			
Marital status	Other	29 (38.16)	47 (61.84)	Chi square	1.971	0.373
	Unmarried	197 (30.83)	442 (69.17)			
	Married	1,025 (32.68)	2,111 (67.32)			
Do you have siblings	No	239 (31.41)	522 (68.59)	Chi square	0.503	0.478
	Yes	1,012 (32.75)	2,078 (67.25)			
Do you have children?	No	303 (32.90)	618 (67.10)	Chi square	0.095	0.758
	Yes	948 (32.35)	1,982 (67.65)			
Medical working time	0–5 years	218 (30.97)	486 (69.03)	Chi square	10.168	0.017
	11–15 years	268 (33.71)	527 (66.29)			
	6–10 years	363 (35.83)	650 (64.17)			
	Over 15 years	402 (30.02)	937 (69.98)			
Professional title	Primary	425 (33.70)	836 (66.30)	Chi square	12.595	0.006
	Intermediate	491 (34.58)	929 (65.42)			
	Deputy senior	230 (29.60)	547 (70.40)			
	Senior	105 (26.72)	288 (73.28)			
Occupation	Nurse	531 (34.77)	996 (65.23)	Chi square	6.045	0.014
	Doctor	720 (30.98)	1,604 (69.02)			
Department	other	283 (29.03)	692 (70.97)	Chi square	7.124	0.008
	ICU	968 (33.66)	1,908 (66.34)			
Religious belief	No	1,177 (32.50)	2,444 (67.50)	Chi square	0.011	0.917
	Yes	74 (32.17)	156 (67.83)			
Participated in public health emergency treatment before	No	861 (32.33)	1,802 (67.67)	Chi square	0.092	0.761
	Yes	390 (32.83)	798 (67.17)			
Directly participate in COVID-19 anti-epidemic work	No	971 (36.77)	1,670 (63.23)	Chi square	70.247	<0.001
	Yes	280 (23.14)	930 (76.86)			

### SCL-90 Score and Positive Symptom Rate of ICU Practitioners

The Cronbach's alpha of every factor was over 0.85, indicating the high reliability and validity. The mean SCl-90 score of all the participants in this survey was 147.84 ± 58.45. Compared with the Chinese norm, the scores of eight factors of SOM, OC, DEP, ANX, HOS, PHOB, paranoid ideation, and PSY of the male and female ICU practitioners were both higher than those of the norm except for IS (*P* < 0.001). In terms of IS, the male and female ICU practitioners were different by comparison with the norm, with no significant difference was found in males (*p* = 0.735). By contrast, the score of female ICU practitioners was still higher than the norm (*p* < 0.001). The mean positive number of the 90 symptoms among ICU practitioners was 34.57 ± 27.90, which was also significantly higher than the Chinese norm population. The results are shown in Table 1.

According to the total score of SCL-90, the overall positive symptom rate of ICU practitioners was 32.49% (95% CI: 31.01–33.96). Unifactorial analysis revealed that women, intermediate education (bachelor's degree), intermediate working time (6–15 years), lower professional title, nurse occupation, being from ICU, and those who did not directly participate in COVID-19 epidemic had higher positive symptom rate (*p* < 0.05), as shown in Table 2. The characteristics of ICU practitioners were taken as independent variables, and the factors affecting positive symptoms of SCL-90 score were selected by stepwise logistic multivariate analysis, including education background, professional title, department, whether participating in the treatment of public health emergencies, and whether directly participating in antiepidemic work. The risk of positive symptoms of the SCL-90 score increased by 98% (OR = 1.98, 95% CI, 1.682–2.331) among those who did not directly participate in the antiepidemic program. The symptoms of those who directly participated in the antiepidemic program were all less severe in the nine factors, including SOM, OC, IS, DEP, ANX, HOS, PHOB, paranoid ideation, and PSY, as displayed in Table 3.

**Table 3:** Comparison of SCL-90 scoring factors between anti-COVID-19 group and non-anti-COVID-19 group.

**Factor**	**Variable level**	**Non-anti-COVID-19 group, *n* = 2,641 (%)**	**Anti-COVID-19 group, *n* = 1,210 (%)**	**Total**	**Test method**	**Statistics (χ^**2**^)**	***P*-value**
Somatization	No	1,955 (74.02)	1,027 (84.88)	2,982 (77.43)	Chi square	57.353	<0.001
	Light	522 (19.77)	136 (11.24)	658 (17.09)			
	Moderate	147 (5.57)	39 (3.22)	186 (4.83)			
	Severe	17 (0.64)	8 (0.66)	25 (0.65)			
Obsessive-compulsive	No	1,404 (53.16)	870 (71.90)	2,274 (59.05)	Chi square	122.052	<0.001
	Light	854 (32.34)	243 (20.08)	1,097 (28.49)			
	Moderate	326 (12.34)	78 (6.45)	404 (10.49)			
	Severe	57 (2.16)	19 (1.57)	76 (1.97)			
Interpersonal sensitivity	No	1,722 (65.20)	969 (80.08)	2,691 (69.88)	Chi square	90.908	<0.001
	Light	684 (25.90)	174 (14.38)	858 (22.28)			
	Moderate	216 (8.18)	56 (4.63)	272 (7.06)			
	Severe	19 (0.72)	11 (0.91)	30 (0.78)			
Depression	No	1,704 (64.52)	948 (78.35)	2,652 (68.87)	Chi square	76.985	<0.001
	Light	665 (25.18)	190 (15.70)	855 (22.20)			
	Moderate	245 (9.28)	59 (4.88)	304 (7.89)			
	Severe	27 (1.02)	13 (1.07)	40 (1.04)			
Anxiety	No	1,910 (72.32)	993 (82.07)	2,903 (75.38)	Chi square	44.643	<0.001
	Light	550 (20.83)	166 (13.72)	716 (18.59)			
	Moderate	161 (6.10)	41 (3.39)	202 (5.25)			
	Severe	20 (0.76)	10 (0.83)	30 (0.78)			
Hostility	No	1,793 (67.89)	989 (81.74)	2,782 (72.24)	Chi square	79.593	<0.001
	Light	582 (22.04)	149 (12.31)	731 (18.98)			
	Moderate	211 (7.99)	59 (4.88)	270 (7.01)			
	Severe	55 (2.08)	13 (1.07)	68 (1.77)			
Phobic anxiety	No	2,180 (82.54)	1,047 (86.53)	3,227 (83.80)	Chi square	10.869	0.012
	Light	357 (13.52)	120 (9.92)	477 (12.39)			
	Moderate	91 (3.45)	39 (3.22)	130 (3.38)			
	Severe	13 (0.49)	4 (0.33)	17 (0.44)			
Paranoid ideation	No	1,971 (74.63)	1,027 (84.88)	2,998 (77.85)	Chi square	51.688	<0.001
	Light	516 (19.54)	133 (10.99)	649 (16.85)			
	Moderate	125 (4.73)	41 (3.39)	166 (4.31)			
	Severe	29 (1.10)	9 (0.74)	38 (0.99)			
Psychoticism	No	2,069 (78.34)	1,038 (85.79)	3,107 (80.68)	Chi square	31.338	<0.001
	Light	459 (17.38)	129 (10.66)	588 (15.27)			
	Moderate	101 (3.82)	38 (3.14)	139 (3.61)			
	Severe	12 (0.45)	5 (0.41)	17 (0.44)			

### The Influence of Working Conditions on SCL-90 Score During Anti-COVID-19 Epidemic

The overall positive rate of SCL-90 for the anti-COVID-19 epidemic ICU practitioners was 23.14% (95% CI: 20.76–25.52), and the lowest positive rate was 15.29% for the succeeding period. The more satisfied the diet and accommodation during the epidemic, the lower the positive symptom rate. During the period of fighting the epidemic, the longer the average weekly cumulative working hours, the higher the positive rate of symptoms, and the positive rate of those working more than 80 h per week reached 39.18%. The rate of positive symptoms was the highest (31.73%) within 2 weeks of participating in the anti-COVID-19 epidemic campaign. The rate was stable (about 20%) within 2–7 weeks, and there was a small increase (25%) over 8 weeks. The rate of positive symptoms was significantly higher when surrounding colleagues had suspected occupational exposure or were infected by occupational exposure (*p* < 0.001). The two kinds of people who thought their risk of being infected in the period not high, that who thought they had received sufficient training of theory and practice on infectious disease protection and treatment. They had significantly lower positive symptom rate than others, as shown in Table 4. The work status of the ICU practitioners participating in the antiepidemic campaign was taken as an independent variable. The factors influencing the positive symptom of SCL-90 score were screened by stepwise logistic multivariate analysis, including current work status, diet, accommodation, infection status of the surrounding colleagues, work infection risk, protection, and treatment training, as shown in Table 5.

**Table 4:** The proportion of positive symptoms with SCL-90 score among the people directly participate in COVID-19 anti-epidemic work.

**Variable**	**Variable level**	**Positive symptom, *n* = 280 (%)**	**Negative symptom, *n* = 930 (%)**	**Test method**	**Statistics (χ^**2**^)**	***p***
Current working status	Rescue work	58 (26.98)	157 (73.02)	Chi square	28.293	<0.001
	Succeeding	74 (15.29)	410 (84.71)			
	Back to work	148 (28.96)	363 (71.04)			
Whether satisfied with the diet during rescue period	Unsatisfactory	20 (51.28)	19 (48.72)	Chi square	61.599	<0.001
	General	138 (33.17)	278 (66.83)			
	Satisfactory	122 (16.16)	633 (83.84)			
Whether satisfied with the accommodation conditions during rescue period	Unsatisfactory	11 (40.74)	16 (59.26)	Chi square	75.183	<0.001
	General	124 (40.00)	186 (60.00)			
	Satisfactory	145 (16.61)	728 (83.39)			
Accumulated working time of the first line of anti-epidemic	1–14 days	66 (31.73)	142 (68.27)	Chi square	15.402	0.004
	15–28 days	52 (22.71)	177 (77.29)			
	29–42 days	53 (17.79)	245 (82.21)			
	43–56 days	54 (20.53)	209 (79.47)			
	>56 days	55 (25.94)	157 (74.06)			
Average accumulated working hours per week during the rescue period	1–40 h	109 (18.44)	482 (81.56)	Chi square	22.985	<0.001
	41–60 h	97 (25.66)	281 (74.34)			
	61–80 h	36 (25.00)	108 (75.00)			
	Over 80 h	38 (39.18)	59 (60.82)			
Duty time of each shift in COVID-19 area during the period of rescue	Within 4 h	23 (21.10)	86 (78.90)	Chi square	9.177	0.102
	4–6 h	90 (20.55)	348 (79.45)			
	6–8 h	67 (21.47)	245 (78.53)			
	8–10 h	38 (31.40)	83 (68.60)			
	10–12 h	34 (25.76)	98 (74.24)			
	12–24 h	28 (28.57)	70 (71.43)			
Is there any suspicious occupational exposure of colleagues around?	No	149 (19.43)	618 (80.57)	Chi square	16.249	<0.001
	Yes	131 (29.57)	312 (70.43)			
Any colleagues who were infected by occupational exposure during work?	No	241 (21.99)	855 (78.01)	Chi square	8.672	0.003
	Yes	39 (34.21)	75 (65.79)			
Views on the risk of infection in the process of working	Unclear	3 (21.43)	11 (78.57)	Chi square	14.193	0.003
	Low risk	35 (15.91)	185 (84.09)			
	Medium risk	94 (21.12)	351 (78.88)			
	High risk	148 (27.87)	383 (72.13)			
Training on prevention and treatment of infectious diseases	Inadequate theory and poor operability	5 (29.41)	12 (70.59)	Chi square	41.458	<0.001
	The theory and operability are general	66 (31.88)	141 (68.12)			
	Sufficient theory and weak operability	70 (36.08)	124 (63.92)			
	Sufficient theory and strong operability	139 (17.55)	653 (82.45)			

**Table 5:** Multi-factor analysis of positive symptom of SCL-90 score of people directly participate in COVID-19 anti-epidemic work.

**Independent variable**	**Independent variable level**	**OR value**	**95% CI min**	**95% CI max**	***P***
Current working status	Rescue work vs. back to work	0.912	0.624	1.333	<0.001
	Succeeding vs. back to work	0.516	0.368	0.725	
Whether satisfied with the diet during rescue period	Unsatisfactory vs. general	2.078	0.955	4.525	0.002
	Satisfactory vs. general	0.615	0.430	0.879	
Whether satisfied with the accommodation conditions during rescue period	Unsatisfactory vs. general	0.546	0.212	1.406	0.002
	Satisfactory vs. general	0.520	0.359	0.754	
Is there any suspicious occupational exposure of colleagues around?	No vs. yes	0.656	0.488	0.881	0.005
Views on the risk of infection in the process of working	Unclear vs. medium risk	0.987	0.258	3.771	0.034
	Low risk vs. medium risk	0.767	0.490	1.199	
	High risk vs. medium risk	1.384	1.008	1.901	
Training on prevention and treatment of infectious diseases	Inadequate theory and poor operability vs. sufficient theory and strong operability	1.114	0.360	3.447	0.010
	The theory and operability are general vs. sufficient theory and strong operability	1.413	0.973	2.051	
	Sufficient theory and weak operability vs. sufficient theory and strong operability	1.844	1.271	2.674	

## Discussion

This cross-sectional survey enrolled 3,851 respondents and revealed worse conditions of mental health by SCL-90 scale among ICU practitioners compared with the Chinese norm. Moreover, we found that the overall mental health of the COVID-19 group was better than that of the non-COVID-19 group by the SCL-90 scale, which might be due to a state of full feelings and sense of responsibility in the face of the epidemic plus the satisfactory diet and accommodation as well as adequate training on prevention and control of infectious diseases.

Previous studies have shown that COVID-19 has an adverse psychological impact on ordinary citizens during the level I emergency response period through the SCL-90 (17). Compared with the general public, medical health workers, including doctors and nurses, working in front-line clinical positions are the main force for hospitals to complete the task of medical security, but also face a higher risk of infection and intense mental pressure during the COVID-19 epidemic.

Compared to the mental health status of the Chinese norm, the ICU practitioners during the COVID-19 epidemic had higher rates of SOM, OC symptoms, DEP, ANX, HOS, terror, paranoia, and psychosis based on SCL-90 score, in both men and women. In terms of interpersonal relationship sensitivity, no significant difference was found in men, but women were found to be sensitive. According to previous studies, results have indicated gender difference, while men tend to be less inter-personally sensitive than women because women typically remember more emotional information than men (18, 19), which may explain this result. The mean positive numbers among the 90 symptoms of ICU practitioners were also significantly higher than the Chinese norm population, indicating that the ICU practitioners face higher risk of mental health problems, which should be given more attention and support by the whole society.

A study on the mental health of medical and nursing staff in Wuhan reported that among 994 medical and nursing staff, 36.9% had subthreshold mental health disturbances, 34.4% had mild disturbances, 22.4% had moderate disturbances, and 6.2% had severe disturbance in the immediate wake of the viral epidemic (20). Another online survey showed that the prevalence of DEP, ANX, insomnia, and distress symptoms were 50.7, 44.7, 36.1, and 73.4%, respectively, among frontline healthcare workers in China (21). Based on this study, health authorities, academic institutions and societies in China rapidly developed various measures and responses including, education materials and programs, 24 h hotline services, on-site crisis psychological interventions, and relevant research (12). Consequently, the prevalence of psychiatric problems among frontline healthcare workers gradually declined in subsequent studies (22).

In the current study, both unifactorial and logistic multivariate analysis showed that educational background, professional title, department, and whether directly participating in antiepidemic work could likely contribute to a higher positive symptom rate. The risk of positive symptoms of the SCL-90 score increased by 98% among those who did not directly participate in the antiepidemic program. Moreover, the symptoms of those who directly participated in the antiepidemic program were all less severe in the nine factors. The reasons for the psychological distress of medical health workers might be related to the many aspects during COVID-19 epidemic, such as insufficient understanding of the virus, the lack of prevention and control knowledge and equipment, the long-term workload, the high risk of exposure to patients with COVID-19 (23, 24), and the exposure to critical life events (25), such as death. However, from the results, we found that the mental health status of those who directly participated in the antiepidemic was not more severe than those who did not, but was even better. This is not consistent with our hypothesis before the investigation. We assume this could be explained by the following explanations: first, during our investigation period, the domestic epidemic had been basically at a steady stage. Many front-line personnel had returned to their original posts, or even though they were still working in the front-line, the most severe stage had already passed, and their psychological state was relaxed to varying degrees. Second, the mentality of those who voluntarily participated (most of whom were Party members) in the rescue work was strong and well-prepared. Third, those medical workers who participated in the rescue work got enough training about the knowledge of COVID-19 and received sufficient protection equipment. Indeed, no doctor (out of 40,000 medical personnel) from other provinces was infected with COVID-19 during their aid period in Hubei Province (26). Finally, a strong sense of social responsibility and encouragement from the whole society and family played spiritual pillars, which supported them to overcome fear and hesitation, leaving them staying in a healthier mental status. Other incentives or policies from government and institutions may act as a supportive factor in improving their mental health.

Many factors affect the positive symptom rate for participants in the epidemic. From the study, we found that the more the doctors were satisfied with the diet and accommodation, the less likely they would develop positive symptoms. In addition, the average weekly cumulative working hours is also correlated with the rate of positive symptoms. These are in accordance with the results we expected. The rate of positive symptoms was significantly higher when surrounding colleagues had suspected occupational exposure or were infected by occupational exposure. Medical health workers might worry about being infected because different workplaces require different medical skills to tackle different medical conditions. In addition, multivariate analysis screened many factors, including current work status, diet and accommodation conditions, infection status of surrounding colleagues, work infection risk, protection and treatment training, that could influence the SCL-90 score of positive symptom. The results indicate that Chinese government and medical institutions should make various efforts to reduce the pressure on medical and nursing staff, such as sending more medical and nursing staff to reduce work intensity, adopting strict infection control, providing personal protective equipment, offering practical guidance as well as improving their working environment.

This study has some limitations. First, a cross-sectional design was applied to investigate the short term mental health influence of COVID-19; however, long term impact, especially posttraumatic stress disorder, might occur as the COVID-19 proceeds. Second, psychological assessment was only based on online survey and self-reporting tool, and there may be some deviation. Moreover, WeChat is adopted as the tool to distribute questionnaire, where selection, reporting and response bias may exist. At last, our survey was made in the basically stable stage of COVID-19 epidemic in China, and mental health status was only at a moderate level, and it was not for those who were at higher risk.

In conclusion, the overall mental health status of the ICU practitioners is worrying. In addition, among the ICU practitioners, the mental health status in the COVID-19 group is better than that of the non-COVID-19 group, and the reasons may vary. Moreover, for the medical workers in the COVID-19 rescue operation, we should select those who have enough related experience and provide them adequate health protection training and better working conditions to enhance their resilience and psychological well-being.

## Data Availability Statement

The original contributions presented in the study are included in the article/supplementary materials, further inquiries can be directed to the corresponding author.

## Ethics Statement

The studies involving human participants were reviewed and approved by Ethical Committee of Beijing Tongren Hospital. Written informed consent was obtained by all study participants.

## Author Contributions

WC, WH, and DZ conceived and designed the experiments. WC, WH, XL, SK, and LZ performed the experiments. WH, WC, TP, XW, and RR analyzed the data. WC and WH wrote the paper. WC and DZ revised the paper. All authors had reviewed and agreed on the contents of this paper.

## Conflict of Interest

The authors declare that the research was conducted in the absence of any commercial or financial relationships that could be construed as a potential conflict of interest.

## Publisher's Note

All claims expressed in this article are solely those of the authors and do not necessarily represent those of their affiliated organizations, or those of the publisher, the editors and the reviewers. Any product that may be evaluated in this article, or claim that may be made by its manufacturer, is not guaranteed or endorsed by the publisher.
